# Distance-based functional criticality in the human brain: intelligence and emotional intelligence

**DOI:** 10.1186/s12859-021-03973-4

**Published:** 2021-01-26

**Authors:** Lili Jiang, Kaini Qiao, Chunlin Li

**Affiliations:** 1 CAS Key Laboratory of Behavioral Science, Institute of Psychology, Beijing, China; 2 Lifespan Connectomics and Behavior Team, Institute of Psychology, Chinese Academy of Sciences, Beijing, China; 3 Department of Psychology, University of Chinese Academy of Sciences, Shijingshan, Beijing, China; 4 Institute of Psychology, Chinese Academy of Sciences, No. 16 Lincui Road, Chaoyang District, Beijing, 100101 China

**Keywords:** Distance, Functional criticality, Intelligence, Emotional intelligence, MRI, Human brain

## Abstract

**Background:**

Anatomical distance has been identified as a key factor in the organizational principles of the human brain. On the other hand, criticality was proposed to accommodate the multiscale properties of human brain dynamics, and functional criticality based on resting-state functional magnetic resonance imaging (rfMRI) is a sensitive neuroimaging marker for human brain dynamics. Hence, to explore the effects of anatomical distance of the human brain on behaviors in terms of functional criticality, we proposed a revised algorithm of functional criticality called the distance-based vertex-wise index of functional criticality, and assessed this algorithm compared with the original neighborhood-based functional criticality.

**Results:**

We recruited two groups of healthy participants, including young adults and middle-aged participants, for a total of 60 datasets including rfMRI and intelligence as well as emotional intelligence to study how human brain functional criticalities at different spatial scales contribute to individual behaviors. Furthermore, we defined the average distance between the particular behavioral map and vertices with significant functional connectivity as connectivity distance. Our results demonstrated that intelligence and emotional intelligence mapped to different brain regions at different ages. Additionally, intelligence was related to a wider distance range compared to emotional intelligence.

**Conclusions:**

For different age groups, our findings not only provided a linkage between intelligence/emotional intelligence and functional criticality but also quantitatively characterized individual behaviors in terms of anatomical distance.

## Background

Understanding how the human brain works is a major challenge for neuroscientists and especially computational neuroscientists. The cerebral cortex has been intensively proven to be modularly organized, and different modules have been gradually identified with boundaries that are related to different cognitive functions using functional magnetic resonance imaging [1–4]. Together with interactions between the modules mediated by numerous pathways within the cerebral architecture, scientists have basically accepted the concept that the human brain works as a whole-brain network. DTI and fMRI permit the analysis of cortical structural and functional networks at relatively high spatial and temporal resolution [5, 6]. Consistent with graph theory, many organizational principles of cerebral cortex architecture, such as the existence of network hubs and small worldness, have been proposed [7]. However, these data-driven empirical observations did not abstract a simple and quantitative characterization of brain network organizational principles until Ercsey-Ravasz et al. [8] testified that anatomical distance between cortical regions played an important role in macaque brain network organization. These researchers reported that connection weights exponentially decay with the interareal distance, and a single-parameter random graph model based on this rule successfully predicted numerous features of the cortical network.

Coincidentally, physical distance has also been shown to be an influential factor in human brain architecture using traditional resting-state fMRI techniques. The material and metabolic cost of long distances results in decreased internode associations, and this distance-dependent functional profile has been proposed and tested for approximately one decade. Salvador et al. [9] summarized an inverse square law describing the dependence of functional connectivity on anatomical distance. These distance-dependent observations in human brain functional architecture added evidence to Ercsey-Ravasz’s model and inspired our current studies on distance-based functional criticality of the human brain. In addition, numerous studies have addressed different roles of local and distant functional connectivity discriminated by anatomical distance in healthy and unhealthy brain functional organization: local and distant functional interactions have been proven to indicate cortical hierarchical organization [10]; different distributions of long and short connectivity are key neuropathologies of multiple neuropsychiatric diseases [11–14]. Although local and distant connectivity play important roles in human brain function, no study has integrated them into a single theoretical framework to study human brain functional organization.

Network theory has been shown to be useful in characterizing human brain functional organization rules. The human brain requires the coordination of neural activity across many spatial and temporal scales, ranging from neurons and circuits to large-scale networks. Criticality is scale free and could accommodate this multiscale phenomenon in the human brain. Additionally, studies have demonstrated that the human brain works near criticality to accomplish the transitions of task states [15–17]. By deriving the nonlinear dynamics of human brain equations and the neighborhood hypothesis, we previously proposed vertexwise functional criticality to study criticality in healthy brains and Alzheimer’s Disease (AD) progression [18–20]. Now that distance was a key factor in human brain network organization, in this study, we proposed the distance-based vertexwise Index of Functional Criticality (D-vIFC) instead of the neighborhood-based index. Our new algorithm not only originated from critical theory in nonlinear dynamics but also took distance into account and integrated both local and distant connectivity in the human brain architecture.

Intelligence could predict one’s overall level of career achievement and quality of life. It is a very integrated concept and generally involves verbal, performance and social intelligence [21]. Hence, narrowly speaking, intelligence and emotional intelligence dissociate humans from other animals, and they are the two most well-known traits for personal achievements. Many brain imaging studies have confirmed the importance of the frontoparietal network for intelligence [22, 23]. Compared with intelligence, emotional intelligence has been more debate on its construct with less evidence of brain mechanisms. Emotional intelligence is similar to social cognition [24] and largely associated with the amygdala. Jausovec and Jausovec [21] also reported that intelligence (verbal and performance) and emotional intelligence were related to different electrophysiological signals. However, no fMRI studies have addressed intelligence and emotional intelligence together. Given that the prefrontal cortex was proposed to interplay between emotion and cognition [25, 26], does cognitive intelligence overlap with emotional intelligence in the context of brain mechanisms? Do brain mechanisms of intelligence and emotional intelligence interact with age? Are these mechanisms encoded by functional criticalities of different spatial scales within human brain functional architecture?

In this study, we proposed a new algorithm of rfMRI-derived human brain functional criticality based on interareal distance, D-vIFC. This algorithm used a distance-dependent definition of the boundary of the dominant cluster in human brain functional architecture, combining both local and distant functional connectivity of the human brain. Using 60 datasets, including rfMRI, intelligence and emotional intelligence scores, we aimed to study whether associations exist between functional criticality D-vIFC and behaviors as well as age-related interactions. Additionally, we defined a new measurement of brain connectivity, namely, connectivity distance, aiming to validate the distance-dependent D-vIFC associations of intelligence and emotional intelligence and to provide a quantitative characterization for individual behaviors in psychology.

## Methods

### Participants

Sixty-seven healthy subjects (32 males, aged 18.6–64.3) were recruited from the local community or universities by advertisements. All the participants were invited for a detailed mental health interview using the Mini-International Neuro-Psychiatric Interview. Individuals with a history of major neuropsychiatric illness, head injury, alcohol and drug abuse were excluded. Participants were assessed with the Wechsler Adult Intelligence Scale-4th Edition (in Chinese, WAIS-IV), Schutte Self-Report Emotional Intelligence Scale in Chinese Version (SSEIS), State-Trait Anxiety Inventory, Mental Health Continuum-Short Form, Emotion Regulation Questionnaire, Chinese Perceived Stress Scale, Achievement Motivation Scale, and Self-Control Scale. The institutional review board of the Institute of Psychology Chinese Academy of Sciences approved this study, and written informed consent was obtained from individual participants prior to data acquisition.

### Behaviour measures

The WAIS-IV was used to measure cognitive intelligence. The full-scale intelligence quotient (FSIQ) is a composite score obtained from 10 subtests measuring two components of cognitive abilities: general ability and cognitive proficiency. The general ability index (GAI) comprises two subindices: the verbal comprehension index (VCI) and perceptual reasoning index (PRI). The cognitive proficiency index (CPI) comprises two subindices: the working memory index (WMI) and processing speed index (PSI).

The SSEIS was applied to measure emotional intelligence. It is a valid assessment developed by Schutte et al. [27] and originates from the emotional intelligence model of Salovey and Mayer [28]. The Chinese version of the SSEIS exhibits high reliability and validity and consists of 33 items that are assessed using a 5-point Likert scale to measure four dimensions, including emotion perception, emotion management of the self, emotion management of others, and emotion utilization [29]. Participants were asked to respond to each item: ‘1’ represented ‘not true of me’ and ‘5’ represented ‘very true of me’. Finally, the average score of all items was just the total score of emotional intelligence. The Cronbach’s α in the present study was 0.90.

### MRI imaging

All the MRI images were collected on the 3.0 T GE scanner Discovery MR750 at the Institute of Psychology Chinese Academy of Sciences. All the participants completed a T1-weighted structural MRI scan (eyes closed) with an ABI1_t1iso_fspgr sequence (TR = 6.652 ms; TE = 2.928 ms; FA = 12°; matrix = 256 × 256; slice thickness = 1 mm) and an 8-min resting-state fMRI scan (eyes open with a fixation cross) using a gradient echo EPI sequence ABI1_bold_bw_rest (TR = 2000 ms; TE = 30 ms; FA = 90°; number of slices = 33 (interleaved); slice thickness = 3.5 mm; gap = 0.7 mm; and matrix = 64 × 64).

### Imaging data preprocessing

**Fig. 1:**
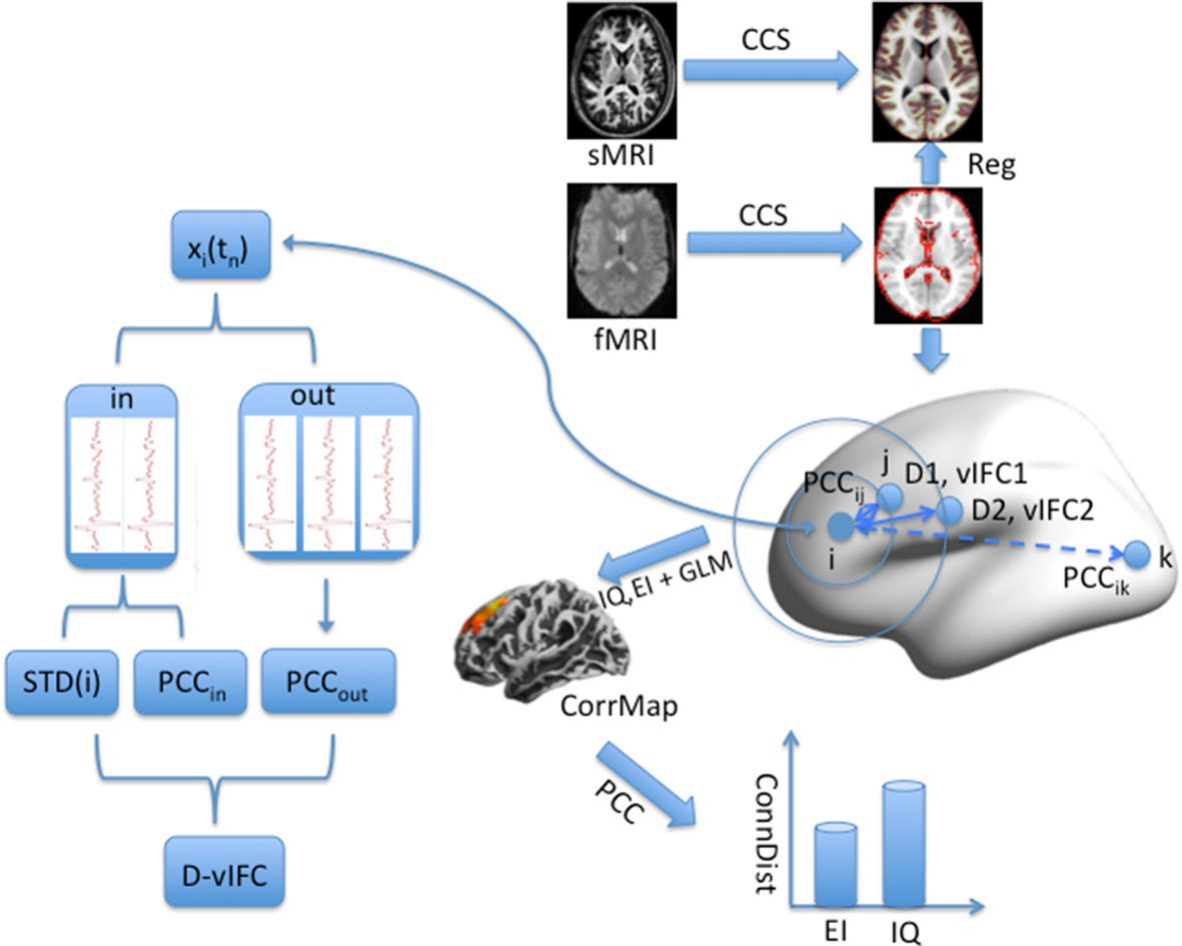
Pipelines for the data analysis strategies used in this study. All the data included structural MRI, functional MRI and behaviors. The data analysis comprised MRI data preprocessing, the vIFC algorithm (the flowchart), GLM statistics and connectivity distance

All the images were preprocessed using the Connectome Computation System (CCS), which was formulated by our lab using FSL, AFNI and FreeSurfer [30]. Its distinctive characteristic is that it focuses on surface-based analysis compared to other resting-state fMRI data analysis pipelines. The system combines anatomical, structural and functional information to provide a computational platform for brain connectome analysis with multimodal neuroimaging data [31]. Preprocessing is composed of structural image preprocessing and functional image preprocessing, and the main purposes of the preprocessing stage are (1) to remove irrelevant brain tissues, including the skull, cerebrospinal fluid, and white matter; (2) to reduce the noise of the MRI images and (3) to facilitate registration across all the participants for the final group analysis. Structural image preprocessing was conducted surrounding cortical surface reconstruction [32, 33], which included (1) T1 image noise removal and brain extraction using the volBrain automated volumetry system (https://www.volbrain.upv.es) [34]; (2) segmentation of cerebrospinal fluid (CSF), white matter (WM) and gray matter (GM), construction of the GM-WM (white surface) and GM-CSF interface (pial surface), and (3) spatial registration by matching of the cortical folding patterns across subjects by recon-all in FreeSurfer. The functional image preprocessing involved more procedures: the first 5 EPI volumes (10 s) are removed to allow for signal equilibration, removal and interpolation of temporal spikes, slice timing correction, alignment of each volume to a ‘base’ volume (the first EPI), normalization of the 4D global mean intensity into 10,000, motion-artifact removal using ICA-AROMA, correction of WM/CSF signals and the Friston-24 motion parameters [35, 36], bandpass (0.01–0.1 Hz) filtering, removal of both linear and quadratic trends, and alignment of the individual functional image to the anatomical image with a GM-WM boundary-based registration algorithm [37]. Finally, individual preprocessed 4D rfMRI time series were projected onto the *fsaverage5* standard cortical surface with 10,242 vertices per hemisphere and an average spacing of approximately 4 mm [38]. Figure 1 shows a simplified pipeline for the data analysis strategy we used in this study.

### Quality control

Quality control plays key roles in making data analysis solid. Here, we considered the following steps for quality control: (1) brain extraction, (2) pial and white surface reconstruction, (3) boundary-based functional image registration, and (4) head motion correction. For the first three procedures, we acquired screenshots and checked their qualities by visual assessment. Quantitative controls of boundary-based functional image registration (mcBBR ≤ 0.65) and head motion (meanFD < 0.3 mm) were also used. One participant did not complete MRI scanning, and one participant did not pass the mental health interview. Five participants were excluded because their mcBBR was greater than 0.65. One participant underwent only the emotional intelligence test but did not undergo the intelligence test. Therefore, 59 participants were included in the intelligence analysis, and 60 participants were included in the emotional intelligence analysis. Considering the wide age range of our participants, we divided all the participants into two groups: the young adult group (aged 19.5–32.8) and the middle-aged group (aged 36.6–64.3 for intelligence and aged 35.9–64.3 for emotional intelligence). Detailed participant information and behavioral measurements are summarized in Tables 1 and 2.

**Table 1:** Participant information. For the young adults, the same participants (17 males and 14 females) were assessed for intelligence and emotional intelligence. For the middle-aged adults, 28 participants were included in the intelligence assessment, and 29 participants were included in the emotional intelligence assessment

	Intelligence	Emotional intelligence
*Young adults*		
Age (years)	26.8 ± 4.3 (19.5–32.8)	26.8 ± 4.3 (19.5–32.8)
Sex (M/F)	17/14	17/14
Edu (years)	16.7 ± 2.5 (9–22)	16.7 ± 2.5 (9–22)
meanFD	0.09 ± 0.03 (0.05–0.15)	0.09 ± 0.03 (0.05–0.15)
errBBR	0.58 ± 0.03 (0.51–0.64)	0.58 ± 0.03 (0.51–0.64)
*Middle-aged*		
Age (years)	50.1 ± 7.6 (36.6–64.3)	49.6 ± 7.9 (35.9–64.3)
Sex (M/F)	14/14	14/15
Edu (years)	14.1 ± 3.3 (8–22)	14.2 ± 3.2 (8–22)
meanFD^a^	0.13 ± 0.06 (0.06–0.29)	0.13 ± 0.06 (0.06–0.29)
errBBR^b^	0.56 ± 0.04 (0.48–0.62)	0.56 ± 0.04 (0.48–0.62)

^a^meanFD is the average of the framewise displacement for in-scanner head motion

^b^mcBBR is the minimal cost of the intrasubject coregistration with the boundary-based registration

**Table 2:** Behavioral measurements of all the participants (N = 60)

Measurements	Average ± STD (Min–Max)
Full-scale IQ	122.83 ± 11.05 (97–142)
General ability index	122.71 ± 11.79 (101–146)
Cognitive proficiency index	119.05 ± 10.95 (92–147)
Verbal comprehension index	123.17 ± 9.92 (107–149)
Perceptual reasoning index	116.71 ± 14.11 (84–144)
Working memory index	115.53 ± 11.89 (89–148)
Processing speed index	117.98 ± 12.57 (92–145)
Emotional intelligence	3.96 ± 0.40 (3.12–4.73)
Emotional perception	3.55 ± 0.46 (2.33–4.50)
Self-emotion management	4.12 ± 0.40 (3.25–5)
Others’ emotion management	4.22 ± 0.54 (2.83–5)
Emotion utilization	4.27 ± 0.56 (2.86–5)

### D-vIFC algorithm

vIFC has been proposed based on nonlinear dynamical theory as an efficient neuroimaging marker that indicates probabilities that a critical transition occurs in the absence of knowledge of the details of realistic network connections [18]. In more detail, vIFC was designed to integrate three properties of the center manifold (subnetwork or a group of variables) in the abstract phase space of the complicated human brain network: increased within-group (dominant cluster) correlations, increased temporal variations and decreased between-group correlations. The neighborhood-based vIFC algorithm has been successfully used in normal and abnormal populations [19, 20]. In this study, we used inter-areal distance instead of neighborhood to define the boundary of the dominant cluster within the entire network architecture. The mathematical formula was the same as the original vIFC and is provided below:

1 $${\text{vIFC}}\left( i \right) = \frac{{STD\left( i \right)PCC_{in} }}{{PCC_{out} }} = \frac{{\sqrt {\mathop \sum \nolimits_{n = 1}^{N} \left( {x_{i} \left( {t_{n} } \right) - \left\langle {x_{i} \left( {t_{n} } \right)} \right\rangle } \right)^{2} } \cdot \left\langle {PCC_{ij} \left( {j \in I} \right)} \right\rangle }}{{\left\langle {PCC_{ik} \left( {k \notin I} \right)} \right\rangle }}$$

As illustrated in Eq. 1, there are three types of vertices, i.e., {i}, $\left\{ {{\text{j}} \in {\text{I}}} \right\}$, and $\left\{ {{\text{k}} \notin {\text{I}}} \right\}$. We chose four distance thresholds (D1 = 14 mm, vIFC1; D2 = 28 mm, vIFC2; D3 = 42 mm, vIFC3; D4 = 56 mm, vIFC4) to explore the associations between vIFC and behaviors at different spatial scales of brain connectivity. Figure 1 depicts D1 (vIFC1) and D2 (vIFC2) as examples. Considering that Sepulcre et al. [10] used 0.25 as a threshold of different distance-based functional connectivity networks, we calculated a Pearson correlation connectivity network for each vertex *i* using connectivity thresholds p_1_ = 0.15, p_2_ = 0.2, p_3_ = 0.25 and p_4_ = 0.3. Then, for given vertex *i*, *I* represents all the vertices that have shorter distances (connecting to *i*) than the distance threshold, *j* represents vertices within *I*, and *k* represents all the vertices that have longer distances (connecting to *i*) than the distance threshold within the entire connectivity network. According to the flowchart in Fig. 1, ‘in’ and ‘out’ represent the vertices inside and outside the sphere, respectively, with the radius of the distance threshold. N is the number of fMRI BOLD time points. x_i_(t_n_) stands for the fMRI BOLD value of the vertex i at time t_n_; PCC stands for the inter-vertex Pearson correlation coefficient across time; STD stands for the standard deviation of the BOLD time series; and <> in the ‘PCC’ means averaging across vertices. The calculation was repeated for each vertex, and we then obtained a D-vIFC map on the fsaverage5 surface of each participant. Considering that the distance thresholds were 14 mm, 28 mm, 42 mm and 56 mm, the vIFC maps were spatially smoothed with Gaussian kernels of 10 mm, 14 mm and 18 mm on *fsaverage5*.

### Statistics

We performed statistical analysis to study the associations between vIFC maps (vIFC1, vIFC2, vIFC3, vIFC4) and behavioral measurements (intelligence and emotional intelligence) as shown in Fig. 1. We employed FreeSurfer Group Descriptor (FSGD) files to generate a general linear model that considered age, sex, and years of education as covariates with DODS (different offset and different slope) settings. For emotional intelligence, we used two different statistical models for its total score (Eq. 2) and subscale scores (Eq. 3):

2 $$vIFC = r_{1} age + r_{2} sex + r_{3} edu + r_{4} EI + e$$

3 $$vIFC = r_{1} age + r_{2} sex + r_{3} edu + r_{4} EP + r_{5} SEM + r_{6} OEM + r_{7} EU + e$$

Similarly, for cognitive intelligence, we used three different models for full-scale intelligence (Eq. 4), two subscales (Eq. 5) and four subscales (Eq. 6) as follows:

4 $$vIFC = r_{1} age + r_{2} sex + r_{3} edu + r_{4} fIQ + e$$

5 $$vIFC = r_{1} age + r_{2} sex + r_{3} edu + r_{4} GAI + r_{5} CPI + r_{6} e$$

6 $$vIFC = r_{1} age + r_{2} sex + r_{3} edu + r_{4} VCI + r_{5} PRI + r_{6} WMI + r_{7} PSI + r_{8} e$$

Finally, the vertex-wise significance values for each contrast of group comparisons were corrected with the false discovery rate (FDR) method (FDR **α** = 0.05/2, corrected *p* = 0.05/2). After acquiring the clusters with significant vIFC-behavior correlations, we also plotted scatters for the residues of the average vIFC within the cluster and behavioral measurements (EI and fIQ) after controlling for age, sex, and years of education.

### Connectivity distance

Distance has been conceived in the algorithm of D-vIFC. Intelligence and emotional intelligence could be related to the functional criticality of different spatial scales. Here, we defined another new measurement called connectivity distance to validate our D-vIFC analysis and further characterize the spatial scales of individual behaviors (Fig. 1). First, we designated clusters with significant correlations between D-vIFC and intelligence (or emotional intelligence) as sigCluster. Then, we calculated whole-brain functional connectivity by taking sigCluster as the seed region for each participant. After Bonferroni multiple comparison correction, we obtained some vertices with significant functional connectivity with sigCluster. Connectivity distance for each participant was defined as the average distance between sigCluster and those vertices with significant functional connectivity. Finally, we obtained the connectivity distance of vIFCi-IQ (i = 1–4, or vIFCi-EI) by averaging the distances of all the sigClusters corresponding to vIFCi versus IQ (EI).

## Results

In this study, we used four smoothing sizes and four thresholds of connectivity (included in the definition of vIFC), and there were 16 sets of results for each statistical analysis. The effects of both smoothing and the threshold of connectivity on the final results were both small and gradually changed from one to one, and this type of consistency indirectly demonstrated the reliability of our algorithm and the analysis used in this study. The threshold of connectivity p_4_ produced pronounced correlations in almost all the analyses in addition to the correlation between vIFC1 and CPI in middle-aged individuals (p_1_). Here, we report the most pronounced results based on p_4,_ 14 mm of spatial smoothing for emotional intelligence and 10 mm of spatial smoothing for cognitive intelligence. Apart from FDR multiple comparison correction of vertex-wise significance and Bonferroni correction for the two hemispheres, we also excluded the results with cluster sizes smaller than 5 vertices.

**Fig. 2:**
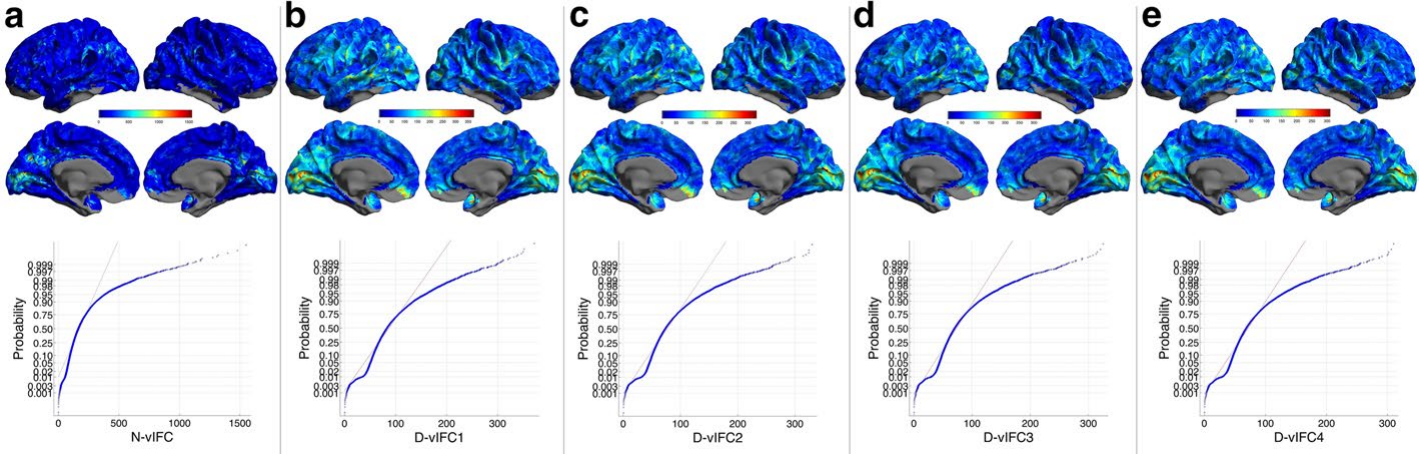
The N-vIFC and D-vIFC of one participant aged 25.6 years across the cortical mantle and their normplots

To provide an intuitive illustration of vIFC variations and a comparison with our previous neighborhood-based N-vIFC, we show the N-vIFC and D-vIFC of four different distance thresholds of one participant aged 25.6 years in the entire cortical mantle in Fig. 2. D-vIFC1, D-vIFC2, D-vIFC3 and D-vIFC4 exhibited very similar profiles. Similar to the original neighborhood-based functional criticality N-vIFC, the inferior parietal and precuneus exhibited large D-vIFCs. The normplots of the D-vIFC values showed that both the left end and the middle of the distribution followed a normal distribution, but only the middle part of N-vIFC followed a normal distribution. This conclusion was verified across all the participants. Our previous algorithm (N-vIFC) was based on a neighborhood in the definition of the ‘within-group’ cluster. The neighborhood only detected very local regions in the cortex, but distance-based vIFC (D-vIFC) could integrate more information at different spatial scales in terms of ‘distance’. D-vIFC values were more similar to a normal distribution and would better characterize individual differences in human brain and behaviors.

### Intelligence and emotional intelligence were related to the functional criticality of separate brain regions for young adults

**Fig. 3:**
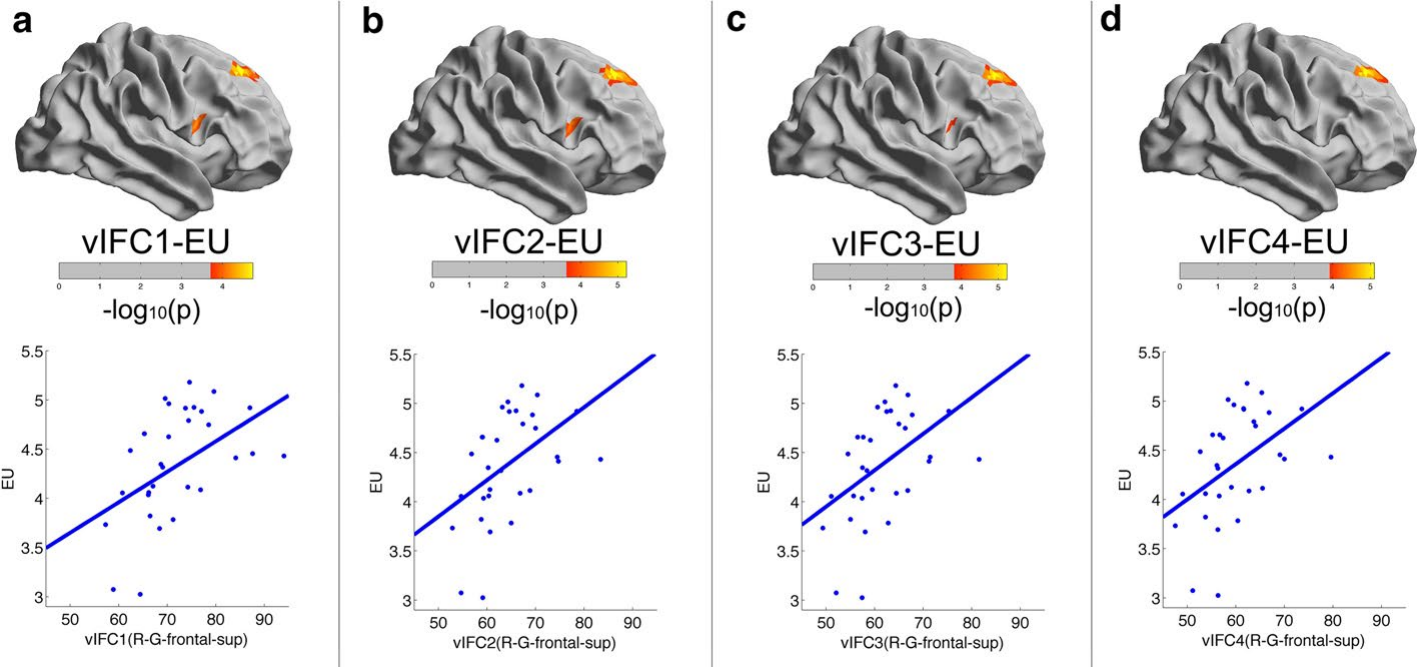
Vertexwise significant positive correlations between vIFC and emotional intelligence across the entire cortical mantle in young adults. Emotion utilization in emotional intelligence increased with vIFC1, vIFC2, vIFC3 and vIFC4 in the right superior frontal gyrus for young adults

**Fig. 4:**
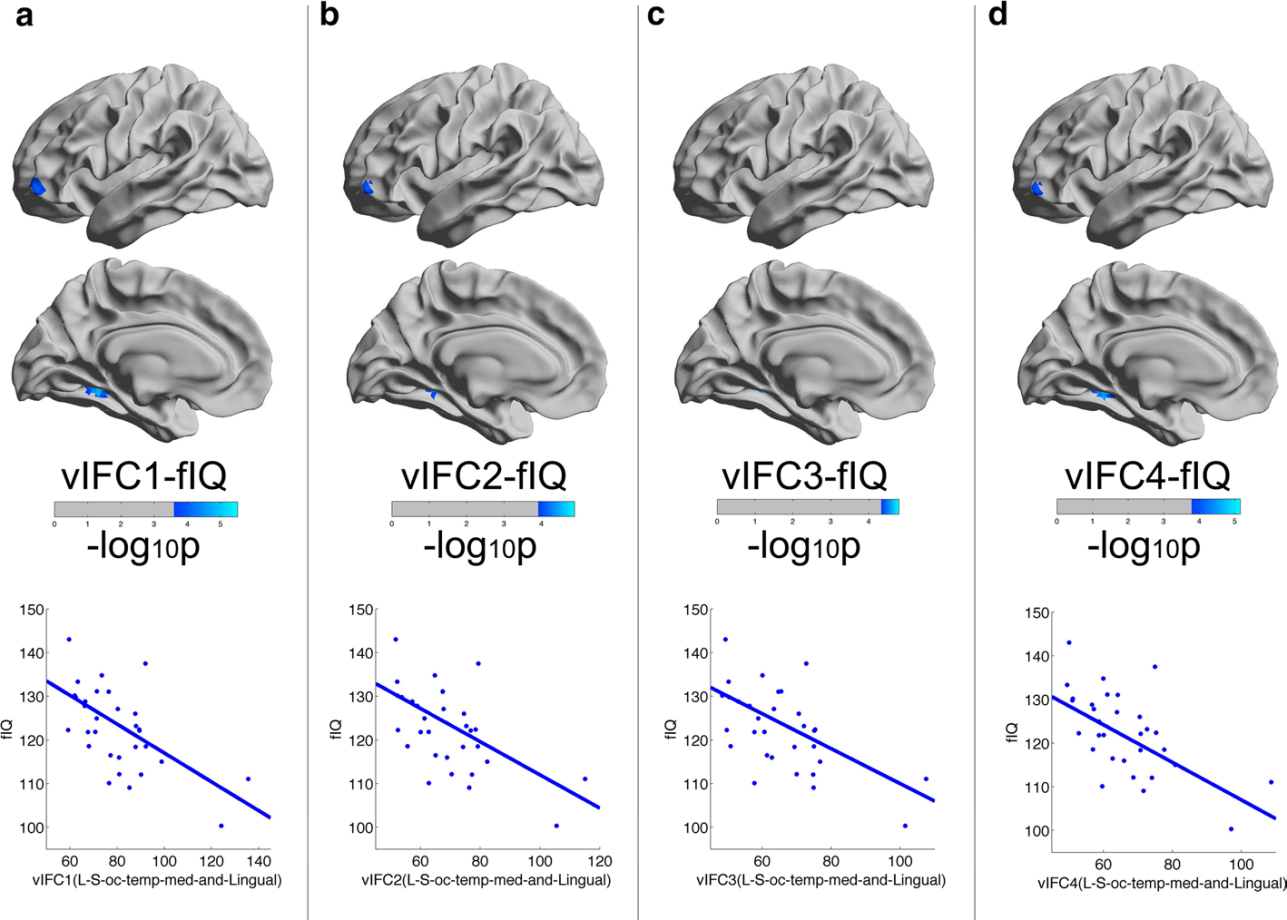
Vertexwise significant negative correlations between vIFC and intelligence across the entire cortical mantle in young adults. Intelligence decreased with vIFC1, vIFC2, vIFC3 and vIFC4 in the left occipital-temporal sulcus in young adults

Figure 3 illustrates vertexwise significant positive correlations between vIFC and emotional intelligence after FDR corrections. Emotion utilization in emotional intelligence increased with vIFC1-4 in the right superior frontal gyrus for young adults. In contrast, intelligence was associated with all four vIFCs. Specifically, intelligence decreased with vIFC1, vIFC2, vIFC3 and vIFC4 in the left occipital-temporal sulcus for young adults, as shown in Fig. 4. Hence, intelligence and emotional intelligence mapped to separate brain regions and did not share any circuits in terms of brain mechanisms. For an intuitive illustration of vIFC at different spatial scales, intelligence and emotional intelligence scores, we also plotted partial correlation scatters for each significant finding as shown in Figs. 3 and 4. Note that we excluded nonsignificant partial correlation clusters in Fig. 3 (the right precentral sulcus) and Fig. 4 (the left orbital gyrus).

### Age-related interactions in brain mechanisms of intelligence and emotional intelligence

**Fig. 5:**
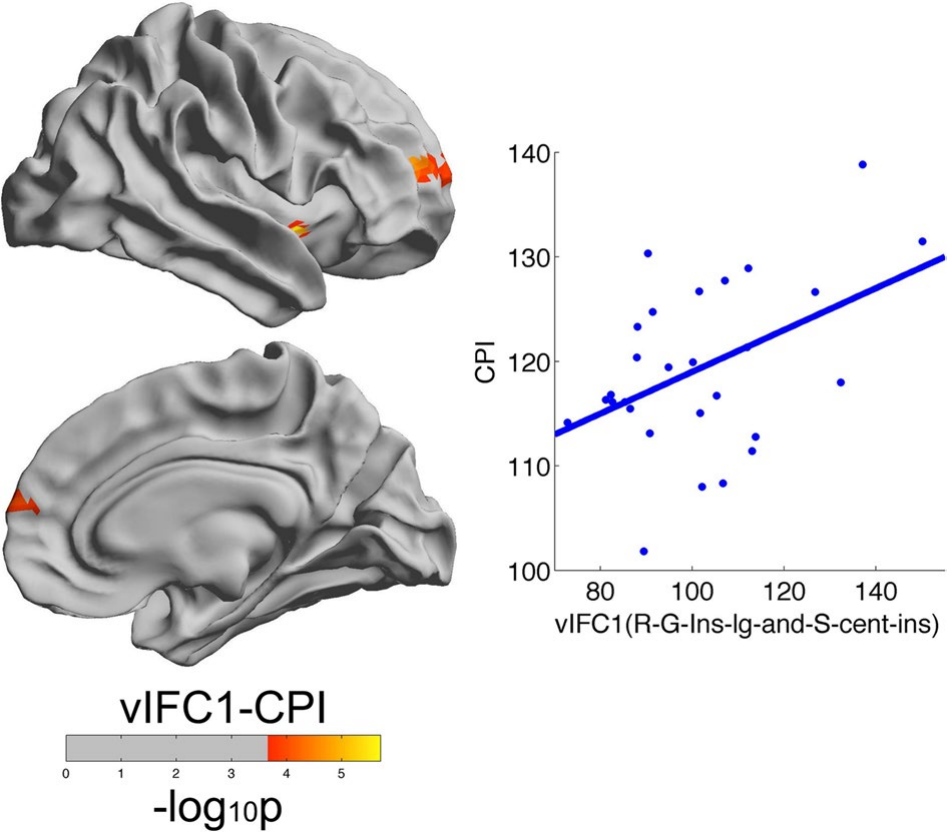
Vertexwise significant positive correlations between vIFC and intelligence across the entire cortical mantle in middle-aged individuals. Cognitive proficiency decreased with vIFC1 in the right insula in middle-aged individuals

Using two groups of different ages, we observed significant age-related interactions in brain mechanisms of intelligence and emotional intelligence. For young adults, we observed significant negative brain-intelligence correlations in the left occipital-temporal sulcus. For middle-aged adults, we observed significant positive brain-intelligence correlations in the right insular gyrus (Fig. 5, the middle frontal sulcus). Additionally, we only observed significant associations of the brain with emotional intelligence in young adults. Such different brain correlates of intelligence and emotional intelligence at different ages indicate different developmental trajectories of intelligence and emotional intelligence.

### Intelligence was associated with a wider distance range than emotional intelligence

**Fig. 6:**
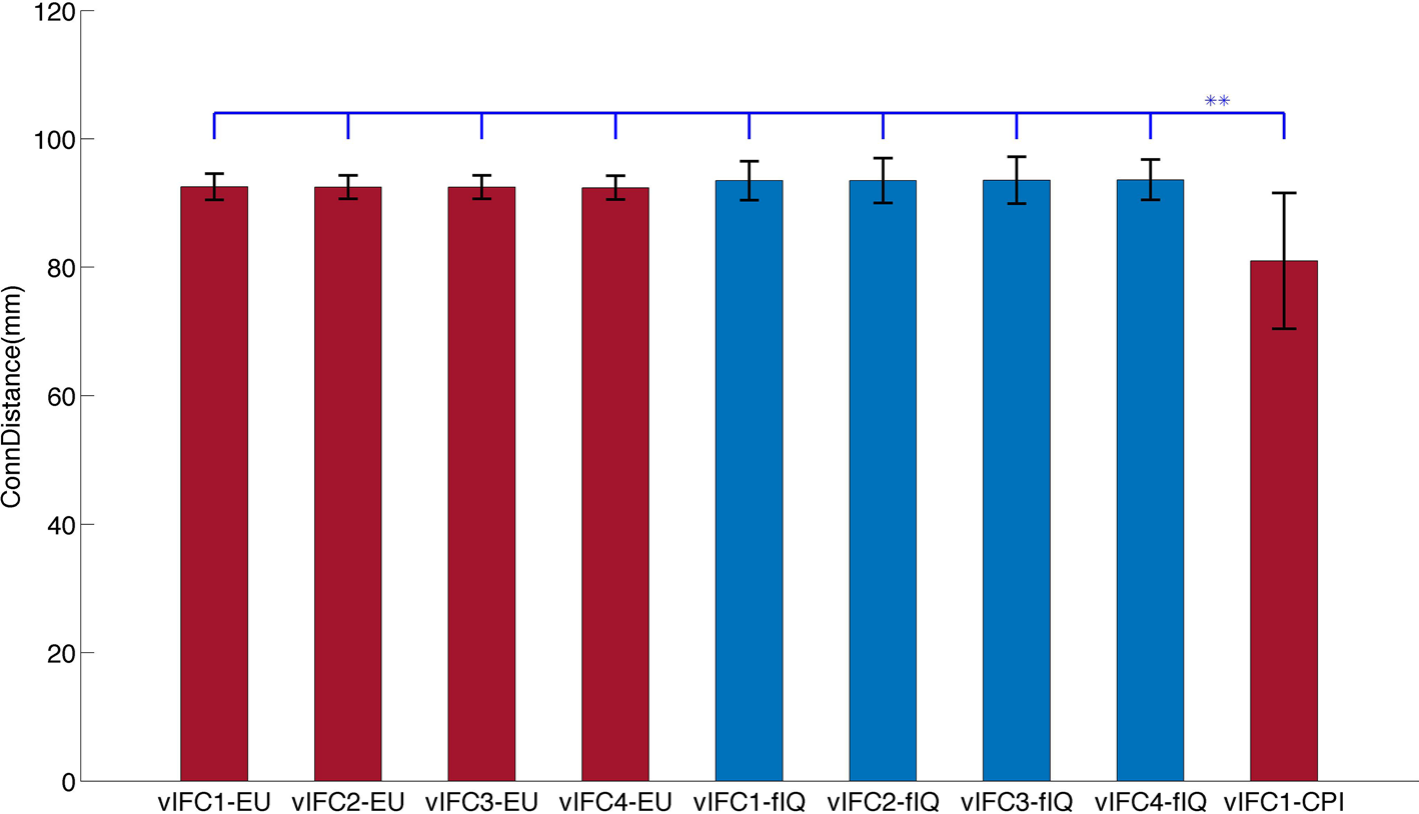
The average connectivity distance for each significant brain-behavior association. Blue and red represent negative and positive correlations of vIFC and behaviors, respectively. We used two stars to represent significant differences at *p* < 0.001 in the two-sample t-test of connectivity distances. Middle-aged participants exhibited shorter connectivity distances of functional criticality compared with young adults

Figure 6 shows the average connectivity distance for each significant brain-behavior association and provides a more direct validation of the differences between intelligence and emotional intelligence. Blue and red represent negative and positive correlations of vIFC and behaviors, respectively. Both emotional intelligence and cognitive intelligence were associated with all distances of functional criticality, vIFC1, vIFC2, vIFC3 and vIFC4, demonstrating that both intelligences were related to multiple spatial scales of brain functional organization. Additionally, when we assessed the clusters with significant vIFC-behavioral correlations in the context of the Yeo-7 network, the right superior frontal gyrus resided in the default mode network. However, the left occipital-temporal sulcus belonged to the visual network, and the right insular gyrus was a part of the ventral attention network. Consequently, intelligence was related not only to a wider distance range but also to more widespread functional hierarchies compared with emotional intelligence. We used two stars to represent significant differences at *p* < 0.001 in the two-sample t-test of connectivity distances. Different thresholds (vIFC1, vIFC2, vIFC3 and vIFC4) contributed to similar connectivity distances; middle-aged participants showed shorter connectivity distances than young adults. When we compared connectivity distances of emotional intelligence with cognitive intelligence, we found that cognitive intelligence had a longer connectivity distance (*p* = 0.002).

## Discussion

Using distance-based functional criticality of the human brain, our study confirmed significant correlations of functional criticality with intelligence as well as emotional intelligence. For young adults, emotional intelligence was positively associated with all distances of functional criticality, and cognitive intelligence was negatively associated with all distances of functional criticality. For middle-aged adults, we observed significantly positive correlations of intelligence with short distance functional criticality. Such different brain correlates of intelligence and emotional intelligence at different ages may indicate different developmental trajectories of intelligence and emotional intelligence. From the perspective of connectivity distance, intelligence was related to a wider distance range than emotional intelligence.

### Intelligence and emotional intelligence were related to distance-based functional criticality of separate brain regions for young adults

In this study, we observed significant correlations of distance-based functional criticality with intelligence and emotional intelligence in separate brain regions. This observation not only demonstrated that both intelligence and emotional intelligence were related to critical transitions of human brain dynamics, again verifying vIFC as an efficient neuroimaging marker [19, 20], but also implied possible interactions of intelligence and emotional intelligence in terms of their brain mechanisms. First, intelligence was a hot topic even 100 years ago, and it has been related to brain size [39, 40], brain morphology [41, 42], brain functional measurements [43–45], and network topology of brain morphology and function, such as network efficiency and the small world [46, 47]. Although the concept of emotional intelligence is not completely accepted, it could represent a good supplement to cognitive intelligence. However, there have been few studies on the associations of intelligence/emotional intelligence with brain dynamics. Compared with neuronal avalanches mediated by structural heterogeneity of networks [48], functional criticality measures the probability of critical transitions of human brain dynamics at the spatial–temporal scale of millimeters and seconds acquired from fMRI. The correlations of intelligence/emotional intelligence with such abrupt changes in human brain dynamics may underlie the contributions of large amounts of neurons spiking to cognition and emotion. In addition, neighborhood-based vIFC has been successfully used to verify MCI as a transition state during AD progression [19] as well as associations with behavioral and physiological measurements [20]. Integrating important topological information distance and eliminating the limit of the neighborhood hypothesis, this study again confirmed functional criticality as an efficient and sensitive neuroimaging marker, thereby promoting its application in future studies on normal and abnormal populations. Second, for the young adult group, intelligence was related to the left occipital-temporal sulcus, and emotional intelligence was related to the right superior frontal gyrus. The occipital-temporal area was sensitive to the face [49], body [50], written Chinese [51], sustained visual attention [52] and multisensory integration [53]. These findings may be related to the comprehension capability of words and figures in intelligence tests. The frontal gyrus is part of the default mode network and is related to task switching [54] and cognitive control [55]. These were all higher-order cognitions and could be used to explain the comprehensive competence in the emotional intelligence test. If there were some overlapping brain regions for intelligence and emotional intelligence, the interactions between them could be explained. Previous studies have demonstrated some interactions between cognition and emotion [25, 26, 56]. However, our results did not show any overlapping regions here. The following reasons may explain these findings. There may indeed be very limited direct interactions between intelligence and emotional intelligence in terms of functional criticality. In addition, our sample size was too small and the number of participants should be increased to deduce more conclusions.

### Age-related interactions in brain mechanisms of intelligence

First, we observed significant age-related interactions in the associations of functional criticality with intelligence. For young adults, intelligence was significantly negatively correlated with functional criticality in the left occipital-temporal sulcus. For middle-aged adults, intelligence was significantly positively correlated with functional criticality in the right insular gyrus. The inverse correlations of functional criticality with intelligence at different ages may be explained as follows. Young adults need fewer large changes in brain activity or metabolic cost to achieve cognitive intelligence; however, middle-aged adults require more large changes in brain activity or metabolic cost to achieve cognitive intelligence. Additionally, the occipital-temporal sulcus is more related to visual function [49–52], and the insular gyrus is more related to limbic and higher-order hierarchy across the entire cortex [57, 58]. These findings consistently indicated that the intelligence of middle-aged adults recruited more resources and higher hierarchy regions compared with young adults, and this conclusion was consistent with the developmental theory of the human brain: the more complicated brain regions, such as the prefrontal cortex, mature the latter [59]. Second, for young adults, intelligence was significantly correlated with all distance-based vIFCs. However, for middle-aged adults, intelligence was significantly correlated with short distance-based vIFCs. Such different distance dependence at different ages was consistent with previous human brain functional development studies that demonstrated that long-range connectivity was more sensitive to aging [60, 61]. Third, there were studies addressing age-related interactions in the associations of amygdala-frontal connectivity with emotional face processing [62]; there were also studies reporting age interactions in the associations of intelligence with electrical signals of the human brain [63] and reinforcing age correlations of intelligence with human brain morphology [64] and human brain functional connectivity [65]. However, no intelligence-related study has demonstrated inverse associations of intelligence with the human brain at different ages. Our study is the first to show a transition from a negative to a positive association of the human brain with intelligence. Actually, not only the development of intelligence and emotional intelligence [66] but also the associations of intelligence with the human brain are dynamic. Our study promotes the use of more specific age bins and gives full consideration to age effects in future human brain studies.

### Intelligence was related to a wider distance range than emotional intelligence

Our study showed that intelligence was related to all distance-based vIFCs in young adults and short-distance vIFC1s in middle-aged adults, but emotional intelligence was only related to all distance-based vIFCs in young adults. Actually, there were only a few studies integrating intelligence and emotional intelligence together [21, 67, 68], and they only involved behavioral measurements but no brain mechanisms. Intelligence is a widely accepted and widely used construct in scientific research, whereas the concept of emotional intelligence is not widely accepted in the academic field. Intelligence comprised reason, plan, solve problem, think abstractly, comprehend complex ideas, learn quickly and learn from experience. Hence, intelligence is a very complicated and complex concept. In contrast, emotional intelligence refers to social intelligence, and the notion of social intelligence as a dark intelligence [69], its dimensional structure [70], and its ability to moderate the relationship between stress and mental health have been assessed [71]. Given the definitions of intelligence and emotional intelligence, intelligence should be the basis of emotional intelligence. Therefore, intelligence was related to wider distance vIFC compared to emotional intelligence.

Furthermore, our study was the first to associate intelligence and emotional intelligence with distance, a quantitative measurement of human brain functional architecture. Intelligence was related to all distance-based functional criticality. This finding is consistent with findings from previous studies indicating that intelligence was associated with shorter characteristic path length [47] together with increased local information processing[72], long-distance theta coherence between frontal and parieto-occipital areas [73], and phase locking between short-distance regions of the frontal cortex [74]. However, there was no distance-related demonstration of emotional intelligence. Given that distance is a key factor in human brain functional organization, different distance dependences may characterize different psychological measurements, such as intelligence and emotional intelligence. Our study may promote distance as a quantitative marker for future psychological behavioral studies.

There were also some limitations in this study. The sample size has become increasingly important in fMRI studies, and our sample size was not large. Sample size may affect our results here, and in the future, big data should be used to verify the associations of distance-dependent vIFC with intelligence and emotional intelligence. Additionally, considering that criticality is a dynamic characteristic close to phase transition, measuring human brain criticality during the performance of tasks rather than resting-state fMRI would be very promising in future studies.

## Conclusions

Our study confirmed that both emotional and cognitive intelligence were associated with functional criticality. For young adults, emotional intelligence was positively associated with all distances of functional criticality, and cognitive intelligence was negatively associated with all distances of functional criticality. For middle-aged adults, only cognitive intelligence was positively correlated with short distance functional criticality. For different age groups, our findings not only revealed an association between intelligence/emotional intelligence and functional criticality but also quantitatively characterized individual behaviors in terms of anatomical distance.

## Data Availability

https://github.com/jiangatbj/D-vIFC.

## Abbreviations

rfMRI: Resting state functional Magnetic Resonance Imaging
D-vIFC: Distance-based vertex-wise Index of Functional Criticality
DTI: Diffusion Tensor Imaging
fMRI: Functional Magnetic Resonance Imaging
AD: Alzheimer’s Disease
WAIS: Wechsler Adult Intelligence Scale
SSEIS: Schutte Self-Report Emotional Intelligence scale
FSIQ (fIQ): Full-scale intelligence quotient
GAI: General ability index
VCI: Verbal comprehension index
PRI: Perceptual reasoning index
CPI: Cognitive proficiency index
WMI: Working memory index
PSI: Processing speed index
MRI: Magnetic Resonance Imaging
TR: Repetition time
TE: Echo time
FA: Flip angle
EPI: Echo planar imaging
CCS: Connectome Computation System
CSF: Cerebrospinal fluid
WM: White matter
GM: Gray matter
ICA-AROMA: Independent Component Analysis-based automatic removal of motion artifacts
FSL: FMRI Software Library
AFNI: Analysis of Functional Neuroimages
FSGD: FreeSurfer Group Descriptor
DODS: Different Offset and Different Slope
FDR: False Discovery Rate
EI: Emotional intelligence
IQ: Intelligence quotient
mcBBR: Minimal cost of Boundary-Based Registration
meanFD: Mean (average) of Frame-wise Displacement
BOLD: Blood oxygenation level dependent
PCC: Pearson correlation coefficient
STD: Standard deviation
sigCluster: (Statistical) Significant cluster
GLM: General linear model
N-vIFC: Neighborhood-based vertex-wise Index of Functional Criticality
MCI: Mild Cognitive Impairment
